# Obituary

**Published:** 1893-08

**Authors:** 


### Obituary.

JAMES McCANN, M. D., LL. D. —Was born in 1836, now fifty-seven years ago, in Penn Township, Allegheny county, Pa. His father served under Anthony Wayne in the war for American Independence. In early boyhood he passed his summers working upon his father's farm, and the winter months as a pupil of John G. Beatty, who taught him, in addition to the public school curriculum of that time, Latin and the higher mathematics. At about thirteen years of age, his father died, when he began teaching school, and became a leading member of a local debating society. Even at this early date he was a ready, fluent, and earnest talker. Tiring of the monotony of country life, and like the majority of young men of his age, not knowing exactly what to do with himself, he decided upon a mercantile career, and at the age of eighteen came to Pittsburgh, where, after graduating at Duff's College, he spent several years as book-keeper in a business house. This sedentary life became irksome to him, his health was not good, and acting upon the advice of his physician, who regarded him as a young man of promising talents, he finally decided to study medicine. With this object in view, he, in 1858, entered the office of Drs. Thomas and John Dickson, Sr.

He graduated from the Medical Department of the University of Pennsylvania in 1863, and immediately entered the medical service of the army as assistant-surgeon of the Fifth Pennsylvania Volunteers. He continued in this service until the close of the war, when he returned to Pittsburgh, and began the practice of medicine with Dr. W. C. Reiter. Two years later he received the appointment of surgeon to the Marine Hospital, and the connection with Dr. Reiter was soon afterward dissolved.

He was next appointed one of the surgeons of the Western Pennsylvania Hospital, which position he held until a few months ago, when he resigned, because of ill-health, and accepted the appointment of Consulting Surgeon. For twenty years he has been one of the surgeons of the Pennsylvania, the Allegheny Valley, and other railroads entering this city.

He was an active and influential member of the Pittsburgh Free Dispensary from its inception, of the Board of Health for many years, of the Allegheny County Medical Society, of the State Medical Society, of the American Medical Association, of the

American Surgical Association, and of the American Association of Obstetricians and Gynecologists, but owing to ill-health he was never able to attend a session of the latter.

In spite of the busy life he led, his ardent love and natural aptitude for teaching led him—in connection with his confreres of the "Mott Medical Club"—to undertake the arduous task of organizing the first medical college in Western Pennsylvania. Into this work he threw all his enthusiasm, and devoted to it all his energy and influence. Caring but little for pecuniary reward, it was with him a labor of love. In September, 1886, after years of weary and distasteful work, the culminating point of his ambition was attained by his election to the chair of Professor of the Principles and Practice of Surgery. This position, notwithstanding his failing health, and in defiance of bodily suffering, he filled until a few months prior to his death. At last, his physical endurance being exhausted, his grateful and sorrowing colleagues unanimously nominated him for appointment as "Emeritus Professor," a last tribute to his eminent worth and ability, but before this action could be confirmed by the Board of Trustees of the University, death had claimed him as his own.

He died a martyr to his profession,—a sacrifice upon the altar of charity. His love for it and devotion to it was the direct cause of his death. He performed an enormous amount of work, and it was in the performance of a surgical operation, a work of charity in the Western Pennsylvania Hospital, that he received the fatal shaft from the quiver of the fell destroyer. Had he, like many others, turned aside from charity work, and devoted himself strictly to his lucrative clientele, he would be living today.

He never ceased to be a student. He was too broad-minded to make a successful specialist. His mental attainments were too great, his studies and reading too comprehensive, his ambition too high for any single department of his profession to permit free scope to his talents. His mind was alert to grasp and tenacious to retain knowledge, which enabled him to easily keep pace with progress and improvement, however rapid, in every department of medical science.

He stood in the front rank of the leaders of the profession. His *savoir faire*, his strong personal individuality, his impulsive and generous nature, won him a host of friends in and out of the profession.

His reputation and practice were not limited to his own city, county, or state, but were national. His life was one of unceasing toil. There are but few surgical operations that he had not performed. His profound knowledge, rather than his personal magnetism, made him popular with all the members of the profession with whom he came in contact, making him eagerly sought as a consultant, and he never betrayed this trust.

He followed, as strictly as the present state of society will perhaps admit, the axiom, "A physician's first duty is to his patient, his second only, to himself."

He was in the active practice of his profession from 1863 to 1893—a period of but thirty years, yet in those thirty years he accomplished, perhaps, a task as great, and fulfilled a destiny as rounded and complete, as the average practitioner of fifty years' standing. A man's life is measured by his works. Judged from this standpoint, although he was but fifty-seven years of age, his death was not premature.

He had faults, but no vices, and his virtues were too many to dwell upon at greater length. By his death his wife loses a loving husband, his children an affectionate father, his colleagues a genial companion and true friend, and his profession a devoted follower. *Requiescat in pace.*

W. SNIVELY,
J. B. MURDOCK,
C. B. KING,
*Committee of Faculty, Western Pennsylvania Medical College.*
PITTSBURGH, June 19, 1893.

